# Sweet tooth: DNA profiling of a cranium from an isolated retained root fragment

**DOI:** 10.1111/1556-4029.14748

**Published:** 2021-06-09

**Authors:** Elena Chierto, Greta Cena, Robert W. Mann, Grazia Mattutino, Emilio Nuzzolese, Carlo Robino

**Affiliations:** ^1^ Department of Public Health Sciences and Pediatrics University of Turin Turin Italy; ^2^ John A. Burns School of Medicine University of Hawaii Honolulu USA

**Keywords:** apical tooth tissue, decalcification, forensic anthropology, forensic DNA typing, forensic odontology, root fragment, skeletal remains

## Abstract

Sampling of healthy multi‐rooted teeth is recommended for the genetic identification of human skeletal remains. However, this may not always be possible, as in the reported case consisting of an isolated human cranium found in an aggregate crushing and processing plant in Piedmont, Northwest Italy. The cranium displayed significant weathering, suggesting a post‐mortem interval of several years, and was edentulous with the exception of the apical root fragment of the upper left canine, consequence of an antemortem horizontal fracture. Prolonged decalcification of the root fragment followed by powder‐free DNA extraction from ~10 mg of root tip tissue led to the recovery of >10 ng of high molecular weight human DNA, in comparison with ~0.01 ng of DNA per mg of bone powder obtained from the petrous portion of the temporal bone. Quantity and quality of DNA isolated from apical tooth tissue enabled multiple genotyping, including a reportable female STR profile, mitochondrial DNA analysis, and ancestry‐informative insertion/deletion polymorphisms. Although the cranium remained unidentified after DNA comparisons, our findings confirm that apical tooth tissue is a promising source of DNA, easily obtained through a powder‐free extraction protocol. Results also indicate that root tips should not be overlooked in challenging identification cases, even in the presence of compromised tooth specimens.

**Highlights**

- A retained dental root was used for DNA identification of a cranium alternative to petrous bone.
- Powder‐free DNA extraction from apical tooth tissue yielded >10 ng of high molecular weight DNA.
- Compromised tooth specimens can be a valuable source of DNA in challenging identification cases.

​

​

## 1 INTRODUCTION

Positive identification of human skeletal remains relies on a combination of methods including forensic anthropology, forensic odontology, and DNA profiling. The mineralized extracellular matrix of bone and tooth guarantees prolonged resistance to exogenous factors of DNA degradation, such as microbial nuclease action and non‐enzymatic degradation, making genetic analysis often successful even in the presence of severely compromised skeletal remains [1, 2].

A general correlation between bone density (i.e. mineralization extent) and DNA profiling success rate was observed in previous studies [3]. Consequently, compact cortical bone from the shaft of long bones, in particular weight‐bearing leg bones, is indicated as the sample of choice in operative guidelines for the identification of human remains [4, 5]. Recent studies have also shown that the inner part of the petrous portion of the temporal bone, being among the hardest bone parts in the mammalian body, can represent a valuable alternative target for genetic analysis, when an isolated cranium is the only remnant found of an unidentified individual [6, 7].

Thanks to their position within the maxillary bones and their unique composition (low porosity and mineralization), teeth are largely protected from the physical and environmental effects that contribute to the peri‐ or post‐mortem degradation process of nucleic acids. For this reason, they can often provide equivalent if not superior DNA yields compared to compact bone samples [8, 9]. Dental pulp is a highly cellular tissue, including mainly odontoblasts and fibroblasts, and represents the most obvious target for forensic DNA typing [10]. However, it has been shown that cellular cementum, predominantly found on the apical segment of the roots and in the furcation area of molar teeth, can also be a valuable source of DNA [11], especially in challenging samples affected by post‐mortem cellular degeneration [12, 13]. For these reasons, the collection of multi‐rooted teeth that provide larger quantities of pulp and cellular cementum is currently recommended in human identification procedures [4, 5]. Since dental caries can cause pulp retraction and, when extensive, complete pulp loss [14], current guidelines also underline the importance to preferably select healthy teeth, without signs of disease or dental restoration, for genetic identification [4, 5].

Despite these caveats, here we report successful DNA profiling of a cranium using the root apex of a retained maxillary canine root as a DNA source, after unsatisfactory genetic analysis carried out on petrous bone.

## 2 CASE HISTORY AND ANTHROPOLOGICAL FINDINGS

**FIGURE 1:**
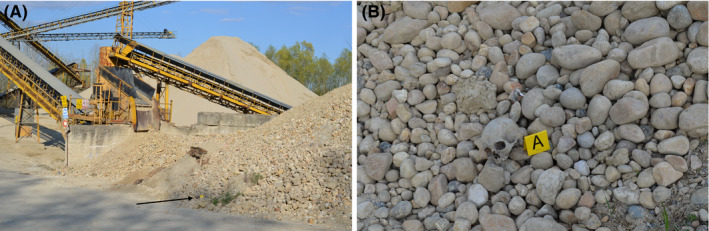
A human cranium was recovered in an aggregate crushing and processing plant in Piedmont (Northwest Italy). Arrow indicates the exact place of discovery (A). A detail is shown in (B) [Color figure can be viewed at wileyonlinelibrary.com]

In April 2020, an isolated jawless cranium was accidentally found lying on a pile of gravel in an aggregate crushing and processing plant in Piedmont, Northwest Italy (Figure 1). The cranium displayed significant weathering compatible with an exposure to the environment of a few years (score 13 according to [15]) (Figure S1). Cranial features were consistent with female sex as confirmed by statistical comparisons of the cranial measurements with samples from Fordisc 3.0.[16] Age assessment according to Meindl and Lovejoy's technique [17] suggested the decedent was middle‐aged (mean age of 52.5 ± 13.5 years). With regard to the determination of ancestry, craniometric evaluation based on 22 measurements performed with Fordisc 3.0 [16] indicated that the individual had the smallest Mahalanobis distance to the Asian female reference group centroid. However, the obtained posterior probability (0.427) and typicality probability (0.001) were too low to support population attribution.

**FIGURE 2:**
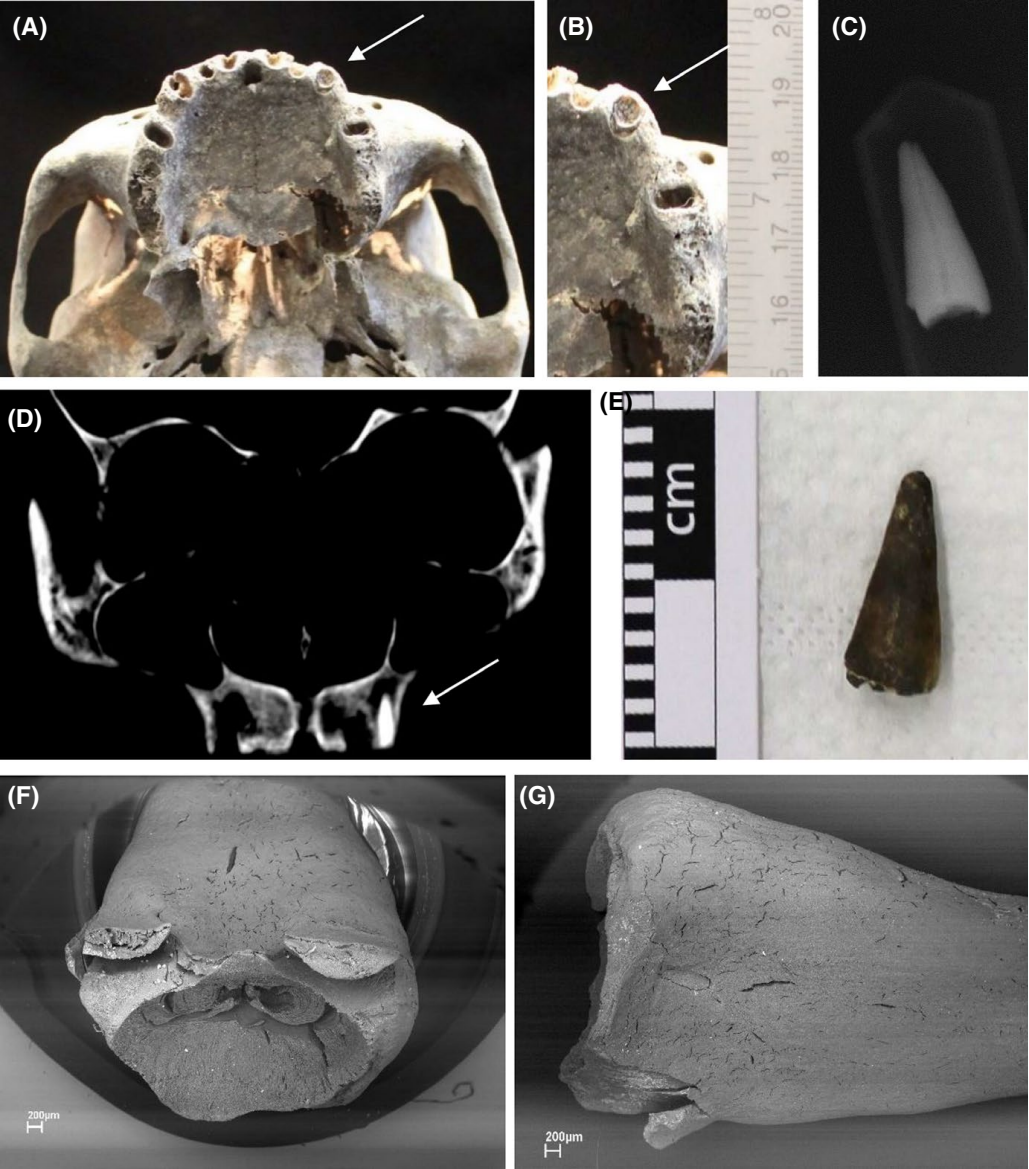
Details of the radicular residue of the upper left canine in situ: inferior view of the cranium (A, B); periapical X‐ray (C); frontal computer tomography scan (D). The radicular residue after detachment from the maxillary bone is shown in (E). Back‐scattered electron (BSE) images obtained with LEO 1430 variable‐pressure scanning electron microscope (SEM) (LEO Electron Microscopy Ldt, Cambridge, UK) of upper (F) and lateral (G) surfaces of the radicular residue show rounded edges indicative of post‐traumatic dental remodeling and ante‐mortem origin [Color figure can be viewed at wileyonlinelibrary.com]

The odontological assessment of the upper jaw (Figure 2) showed it was edentulous, except for the upper left canine (2.3), which consisted of a retained root without the crown due to a horizontal fracture. The root did not present any dental treatment, as confirmed by the periapical X‐ray taken using X‐ray portable device combined with a radiovideography sensor. The magnified observation of the root showed rounded‐off edges indicative of post‐traumatic remodeling and repair [18], demonstrating that the fracture was not peri‐ or post‐mortem

In the subsequent days, further research in the area where the cranium was found led to the discovery of a fragmented left human femur (Figure S1), displaying the same severe weathering process affecting the cranium, compatible with years of exposure to an outdoor environment.

## 3 GENETIC ANALYSIS

### 3.1 DNA extraction and quantitation

In order to prevent contamination, prior to DNA extraction, the outer layers of petrous bone and femur diaphysis were mechanically removed with a rasp. DNA was then isolated from compact bone using PrepFiler BTA Forensic DNA Extraction kit (Thermo Fisher Scientific, Waltham MA, USA) starting from 50 mg of bone powder previously pulverized in liquid nitrogen, according to manufacturer instructions. Two independent extractions were performed from each specimen with a final elution volume of 50 µl.

The radicular residue of the upper left canine spontaneously detached itself from maxillary bone while dissecting the cranium for petrous bone analysis. The whole specimen was washed with 10% bleach, followed by distilled water and 100% ethanol, and then demineralized for 15 days in EDTA (0.5 M pH 8) on a shaker at room temperature, with replacement of the EDTA solution every 48 hours. After demineralization, the sample was cut in serial tissue sections of ~10 mg starting from the root apex and DNA extracted from each section using the QIAamp DNA Investigator kit (Qiagen, Hilden, Germany), following the manufacturer's protocol for isolation of total DNA from tissues (50 µl of final elution volume). All DNA isolation experiments included extraction blanks, which were analyzed in parallel with case samples in the following DNA quantitation and genotyping procedures.

Total and male human genomic DNA isolated from bone and teeth samples was determined by quantitative PCR (qPCR) using the Plexor® HY System (Promega, Madison WI, USA) and CFX96 Touch Real‐Time PCR detection system (Bio‐Rad Laboratories, Hercules CA, USA).

### 3.2 DNA typing

DNA extracts obtained from the cranium (petrous bone, radicular residue of upper left canine) and femur were amplified for the autosomal STR loci included in the PowerPlex ESI 17 Fast kit (Promega, Madison WI, USA) in duplicate reactions. A DNA input of 0.5 ng was used in PCR amplification experiments whenever possible. For extracts with suboptimal DNA concentration, the maximum volume of input DNA according to PowerPlex ESI 17 Fast technical manual (17.5 µl) was included in the reaction. Additional genetic analysis was carried out on DNA isolated from the radicular residue, in order to confirm biogeographic origin and to perform kinship testing with a candidate relative (a male subject missing a sister whose biological profile overlapped that derived from anthropometric measurements of the cranium). This included amplification of Ancestry Informative Markers (AIMs) consisting of a panel of 46 insertion/deletion polymorphisms (Indel) as described by [19]; sequencing of the hypervariable regions HV1 and HV2 of mitochondrial DNA (mtDNA), using primer pairs F15971‐R16410 for HV1 and F15‐R389 for HV2 [20] and the Big Dye Terminator version 3.1 Cycle Sequencing kit (Thermo Fisher Scientific, Waltham, MA, USA).

Detection and separation of PCR (STR, AIM‐Indels) and sequencing (mtDNA) products were carried out using the Seqstudio Genetic Analyzer (Thermo Fisher Scientific, Waltham, MA, USA). Fragment analysis (STR, AIMs) was performed with software GeneMapper 5 (Thermo Fisher Scientific, Waltham, MA, USA). mtDNA sequences (16024‐16365 tract for HV1, 72‐340 tract for HV2) were analyzed with SeqScape version 3 software (Thermo Fisher Scientific, Waltham, MA, USA) in comparison with the human mtDNA reference sequence (revised Cambridge Reference Sequence), in accordance with the dictates of the International Society of Forensic Genetics (ISFG) [21, 22].

STR profiles and mtDNA haplotypes obtained from bone and tooth samples were compared against the laboratory staff elimination database to rule out potential contamination.

### 3.3 Ancestry inference

For the inference of the biogeographical origin through AIM‐Indels, the functionalities contained in the Snipper portal (http://mathgene.usc.es/snipper/index.php) were used [23].

## 4 GENETIC RESULTS

Mean concentration of total DNA in the two petrous bone extracts was 0.011 ng/µl, while the concentration of male DNA was below the range of DNA standards used to set up the quantitation experiment (<3.2 pg/µl). PCR duplicates from the two extracts all displayed female genotypes at the Amelogenin locus, in accordance with anthropological findings. However, STR typing results were of poor quality (Figure S2), with limited replicability of the genotypes due to drop‐out and drop‐in artifacts. The obtained DNA profile was therefore unreportable according to guidelines of the Italian working group (GeFI) of ISFG [24] that require at least 10 replicated loci in independent PCR experiments. Evaluation of Internal PCR Control ΔCt in qPCR experiments excluded that the unsatisfactory STR profiling results could be due to co‐isolation of PCR inhibitors.

Total DNA yield from the root apex section of the canine radicular residue was decidedly higher (0.230 ng/µl) compared to petrous bone. Total DNA concentration was reduced to 0.053 ng/µl in the adjacent tissue section and declined to <3.2 pg/µl in the following tissue sections. The absence of detectable male DNA (<3.2 pg/µl), as observed in petrous bone extracts, was confirmed. Amplification of DNA isolated from the root apex section of the canine radicular residue led to complete and fully replicable autosomal STR profiles, while sporadic drop‐out and drop‐in events at high molecular weight loci (D10S1248, D2S441) were observed in independent amplification reactions of DNA isolated from the tissue section immediately adjacent to root apex (Figure S2). The obtained STR profile was therefore reportable according to GeFI guidelines and suitable for inclusion in the missing person section on the Italian National DNA database. Amelogenin locus genotypes obtained in all PCR replicates confirmed that the cranium belonged to a female subject.

Comparisons between DNA profiles obtained from the root apex and femur extracts (mean total DNA yield 0.044 ng/µl) showed multiple genotype incompatibilities, among them a X‐Y genotype at Amelogenin locus for the femur sample, thus excluding that cranium and femur could belong to the same individual (Figure S2).

**FIGURE 3:**
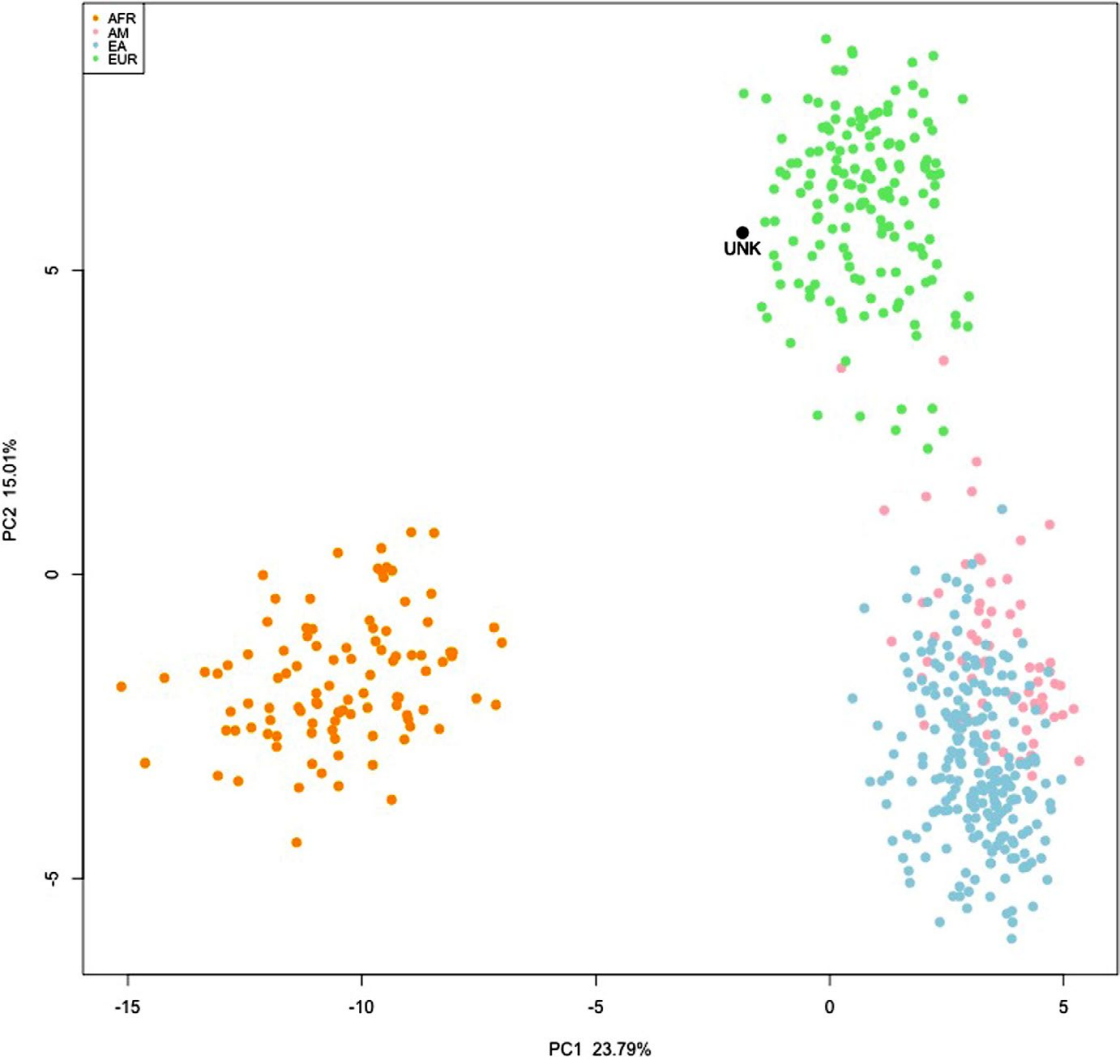
Snipper PCA plot of AIM‐Indels indicating that DNA isolated from root apex (UNK, black dot) clusters with HGDP European reference samples (green dots) compared to sub‐Saharan African (orange dots), Native Americans (pink dots) and East Asian (azure dots) reference populations [Color figure can be viewed at wileyonlinelibrary.com]

Comparison of HV1 and HV2 mtDNA haplotypes obtained from root apex section of the canine radicular residue (73G, 146C, 150 T, 152C, 263G, 295 T, 309.1C, 315.1C, 16069 T, 16126C, 16193 T) and tested candidate (alleged brother of a missing woman) led to an exclusion. The cranium therefore remains unidentified. A search in the forensic mtDNA haplotype database EMPOP [25] did not produce any exact match. The most likely mtDNA haplogroup inferred from mtDNA haplotype according to the maximum likelihood approach implemented in EMPOP [26] was J2b, typical of Europe and Near/Middle East [27]. To further assess biogeographic ancestry with AIM‐Indels, a four population comparison with European, sub‐Saharan African, East Asian, and Native American reference samples from the Human Genome Diversity Project (HGDP) panel [19] was conducted with Snipper. The obtained consensus profile including 44 loci, that is, all those that showed genotype replication in PCR duplicates of DNA extracted from the root apex section of the canine radicular residue, resulted to be >10^9^ times more likely to be European (‐log likelihood 39.4) rather than sub‐Saharan African (‐log likelihood 72.4), East Asian (‐log likelihood 80.4), or Native American (‐log likelihood 76.2). European origin was also confirmed by principal component analysis (PCA), with the cranium sample clustering with HGDP European reference samples (Figure 3).

## 5 DISCUSSION

Despite the recommendation to analyze healthy, multi‐rooted teeth in order to increase the success in DNA analysis [4, 5, 28], there is a growing interest in the study of restored and/or decayed teeth as a source of DNA. This is due to the fact that intact teeth may not be always available in real forensic investigations, as in the present case where an isolated cranium with only a radicular residue of the upper left canine was found.

It was previously shown that teeth with caries, extracted following orthodontic or periodontal disease treatment, can still contain enough pulp tissue to ensure successful STR typing [29]. Pulp, however, undergoes a quick loss of structural integrity post‐mortem, with nuclear DNA yields rapidly declining beyond a four‐month post‐mortem interval (PMI) [30].

On the contrary, successful DNA profiling from cementocytes is often possible even after prolonged PMI and in challenging post‐mortem conditions [12], even in single‐rooted teeth with comparatively smaller pulp volume [13]. Moreover, cellular cementum is largely unaffected by dental caries and periodontal disease [10]. Experimental studies have shown that suitable DNA yields for identification purposes can be obtained from cementum of teeth that underwent root canal treatment and restoration [31], or affected by periodontitis and pulpal/periapical disease [14, 31]. Accordingly, DNA kinship analysis on single‐rooted restored teeth, collected from exhumed human remains buried for 46 years, was recently reported [32].

DNA is routinely isolated from cementocytes after pulverization of whole teeth or root segments [33]. This approach, however, presents several disadvantages [10]: It requires dedicated and expensive instrumentation; the heat generated in the crushing process can affect DNA integrity; and the presence of large amounts of acellular material increases the risk of contamination, dilutes DNA content, and interferes with the following extraction steps. An effective alternative is represented by powder‐free methods in which, after prolonged decalcification of whole teeth, demineralized root tips are collected and submitted to standard forensic DNA extraction protocols designed for soft tissue samples [34, 35]. Preferential targeting of root tips is justified by the higher thickness and cellularity of cementum expected in apical radicular portions [36]. Such methods, besides being simple and inexpensive, are also minimally destructive, so that relevant teeth parts can be preserved for forensic odontology, and the tested samples returned to families after identification. This report indicates that a similar approach can be applied not only to restored teeth with long PMI [32], but also to isolated root fragments. In particular, the root apex was confirmed as an optimal target for DNA isolation, with DNA yields rapidly declining in the adjacent cervical radicular segments.

In this case study, the retained root emerged above the alveolar crest and was therefore an obvious target for molecular analysis. It can be assumed that the upper canine received trauma which caused the transverse fracture of the crown leaving the root *in situ*, followed by incomplete healing and interposition of soft tissue [37]. In such traumatic injuries, the apex of the root is usually unaffected and there is no damage to cementum and periodontal ligament [38]. At the periphery of the fracture line, together with remodeling and resorption of the edges, the formation of new cementum may occur, which may even join the two fragments to some extent [37]. In the present case, however, no detectable DNA could be isolated from the coronal end of the root fragment.

Transverse root fracture is an infrequent type of traumatic dental injury, with prevalence between 0.5 and 7% in permanent teeth and highest incidence in the maxillary anterior region [39]. Root fractures, however, can also occur during teeth extraction. In such cases, root tips can successfully remain *in situ* with normal healing taking place together with the formation of a cementum layer on the dentine allowing bone deposition, thus enclosing the root fragment within bone [40]. While prevalence varies between studies, accidental finding of at least one retained root in panoramic radiographs of edentulous patients is reported with frequency between 9% and 46% [41]. Our results suggest that a careful search for retained roots, taking advantage of radiological methods, should always be performed during dental autopsy, since embedded root fragments can represent a valuable source of DNA in addition to petrous bone in challenging identification cases of isolated, apparently edentulous cranium specimens.

## 6 CONCLUSION

Joint anthropological, odontological, radiological, and genetic investigations indicated that the recovered skeletal remains consisted of a commingling of human specimens, with cranium and femur belonging to two different individuals. While the cranium remains unidentified, it was shown that a single fractured dental root, despite long‐term (exceeding a few years) PMI and tissue modifications due to mechanical damage, can provide high molecular weight DNA at concentrations suitable with forensic analysis of STRs, mtDNA, and AIM‐Indels. A powder‐free protocol previously applied with success to healthy and restored post‐mortem teeth was used to isolate DNA from the demineralized root tip. The method is straightforward, compared to more complex procedures adopted for other skeletal material, its major drawback being the need for prolonged decalcification, making it less suitable for high priority cases that require immediate processing of samples. Obviously, results from this case report cannot be generalized and need to be supplemented in the future by larger comparative studies of DNA yield from intact/damaged post‐mortem teeth and other cranial elements in different taphonomic conditions. If confirmed, persistence of high‐quality genetic material in apical tooth tissue, even in conjunction with trauma, disease, and dental work, will provide forensic investigators with an alternative and easily accessible DNA source, not to be overlooked in the analysis of challenging human remains.

## Supporting information

Fig S1

Fig S2
