# Supplementary material for: Sweet tooth: DNA profiling of a cranium from an isolated retained root fragment
Source: J Forensic Sci. 2021 Jun 9;66(5):1973–9. doi: 10.1111/1556-4029.14748 (PMC8453871; doi:10.1111/1556-4029.14748)
Supplement: Supplementary file 1 — Fig S1 [file JFO-66-1973-s002.pdf]

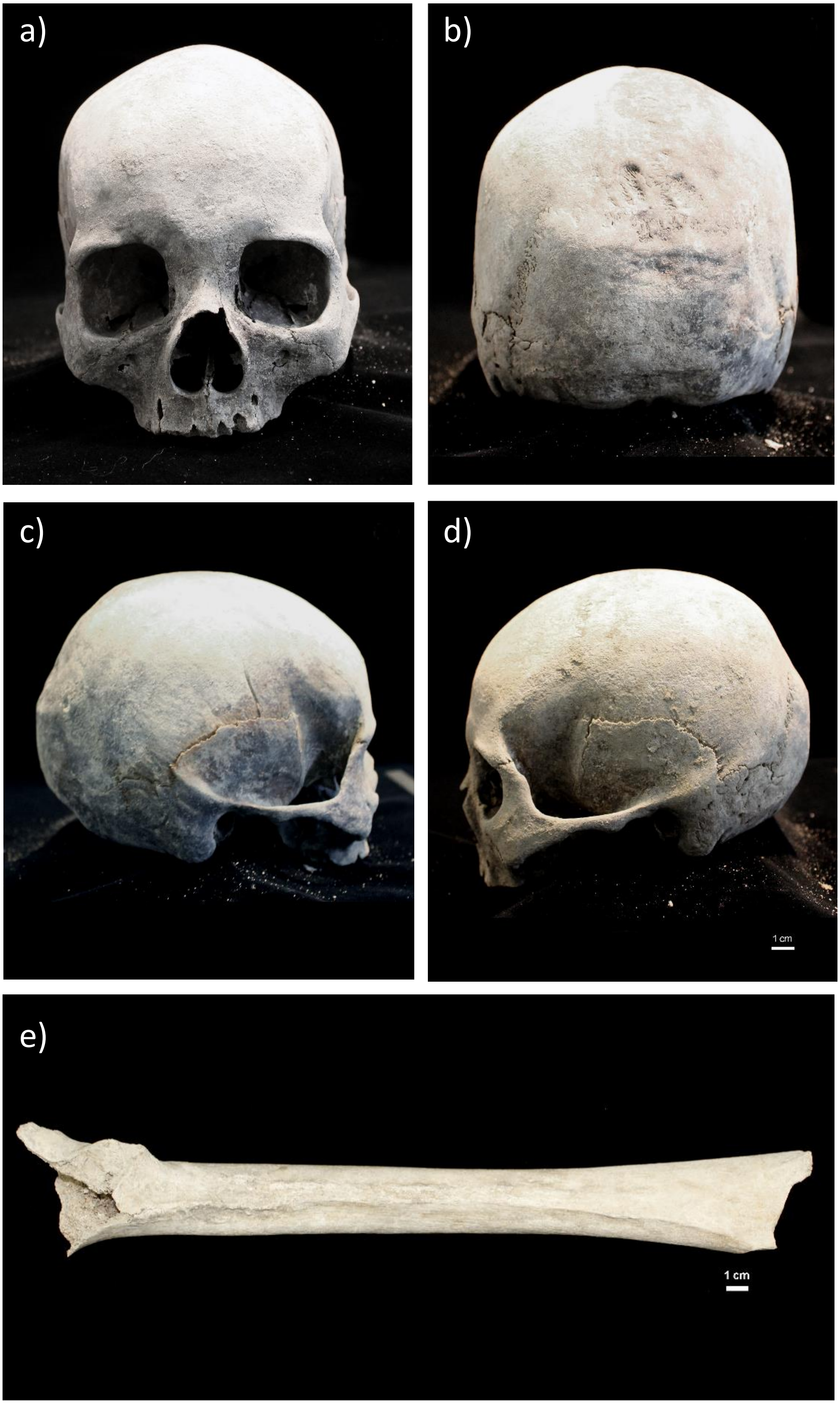

Figure S1 - *Anterior (a), posterior (b), right (c) and left (d) lateral views of the cranium. Left human femur found near the cranium after further research (e).*
