# Supplementary material for: Sweet tooth: DNA profiling of a cranium from an isolated retained root fragment
Source: J Forensic Sci. 2021 Jun 9;66(5):1973–9. doi: 10.1111/1556-4029.14748 (PMC8453871; doi:10.1111/1556-4029.14748)
Supplement: Supplementary file 2 — Fig S2 [file JFO-66-1973-s001.pdf]

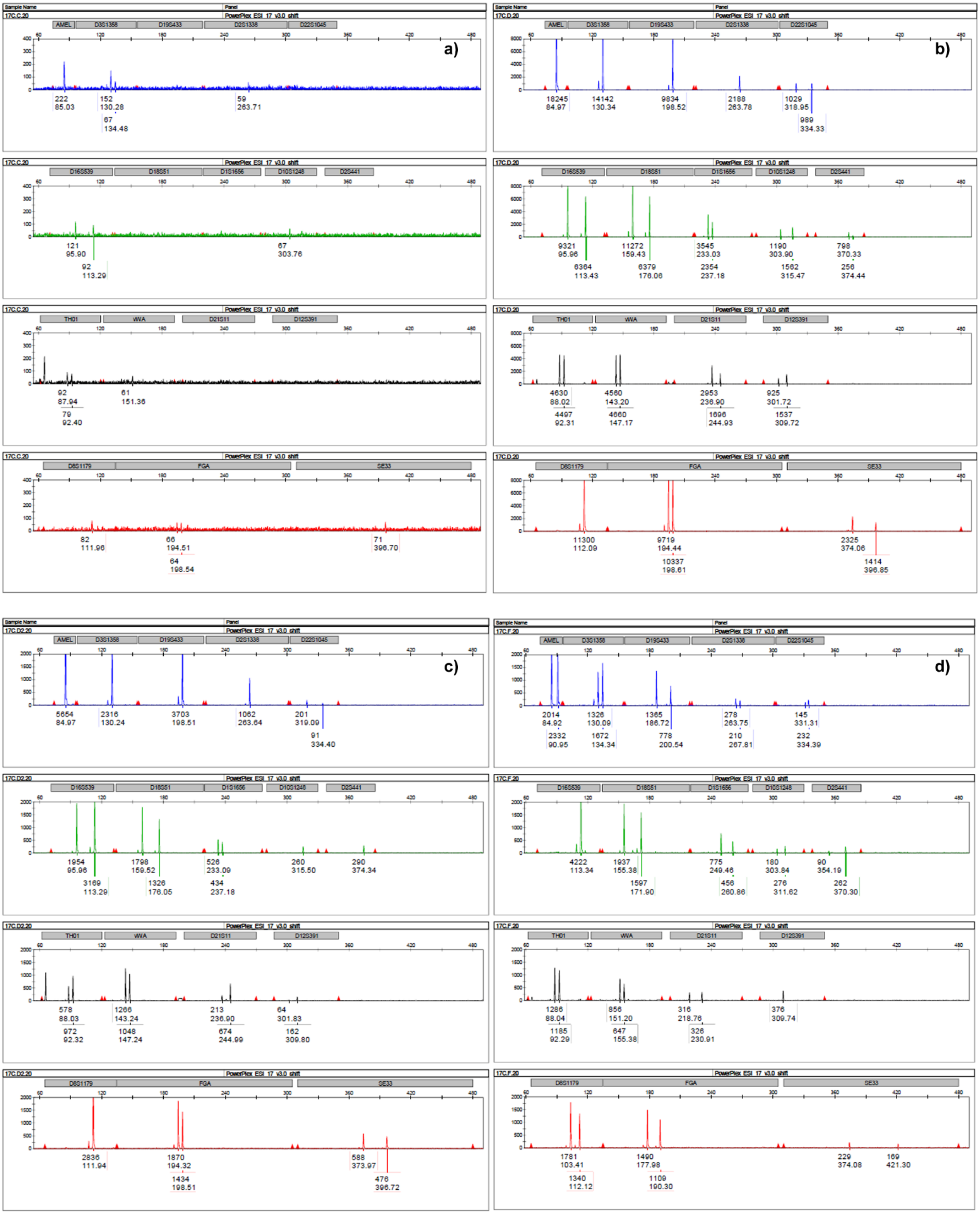

Figure S2 - *A selection of electropherograms obtained from human skeletal remains. DNA profile from the petrous bone of the cranium is shown in a). Results from the radicular residue of the left upper canine are shown in b) (root apex sample) and c) (tissue section adjacent to the root apex). The DNA profile of the human femur is displayed in d). For privacy reasons, allele designations were removed from peak labels. Peak height (rfu) and estimated peak size (bp) are reported.*
